# Supplementary figures and images for: A Bayesian Monte Carlo approach for predicting the spread of infectious diseases
Source: PLoS One. 2019 Dec 18;14(12):e0225838. doi: 10.1371/journal.pone.0225838 (PMC6919583; doi:10.1371/journal.pone.0225838)

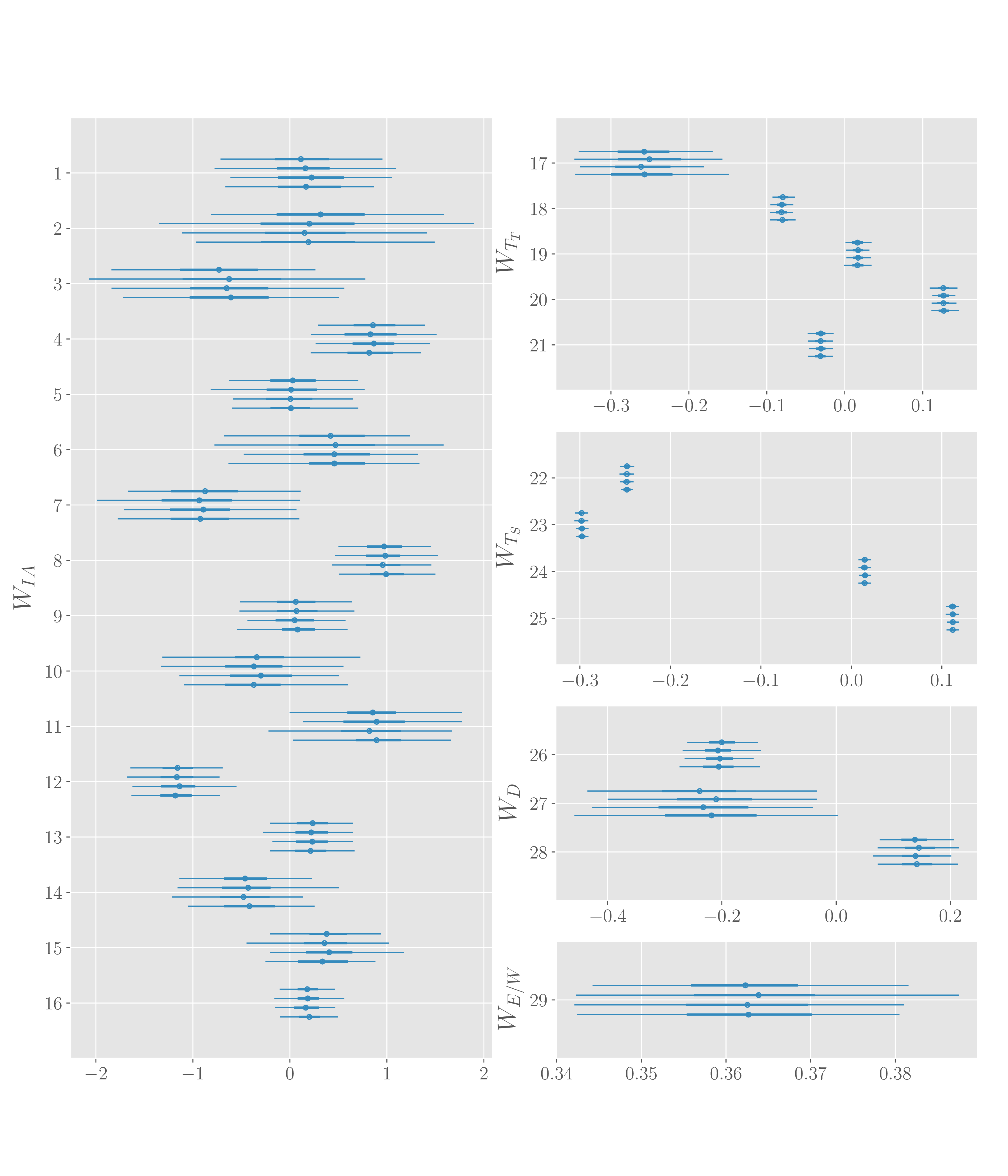

Supplement: S1 Fig — For each of four Markov chains, the mean (dot), the range from the 25% to 75% percentile (thick horizontal lines) as well as the 2.5% to 97.5% percentile (thin horizontal lines) are shown. For all parameters, these summary statistics of the marginal distribution are similar across all four chains, indicating convergence of the MCMC sampling scheme (see also S7 Fig). (TIFF) [file pone.0225838.s001.tiff]

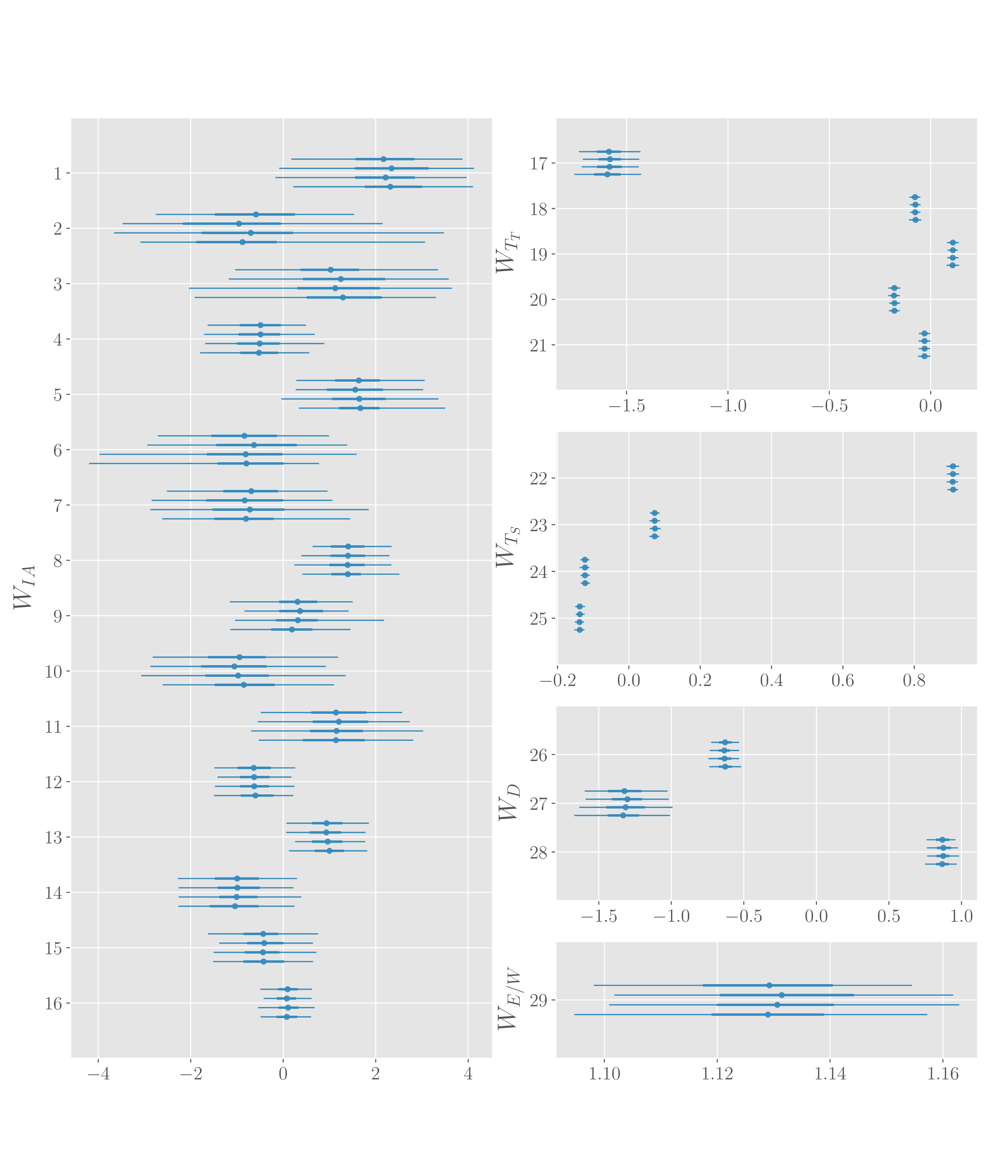

Supplement: S2 Fig — For each of four Markov chains, the mean (dot), the range from the 25% to 75% percentile (thick horizontal lines) as well as the 2.5% to 97.5% percentile (thin horizontal lines) are shown. For all parameters, these summary statistics of the marginal distribution are similar across all four chains, indicating convergence of the MCMC sampling scheme (see also S7 Fig). (TIFF) [file pone.0225838.s002.tiff]

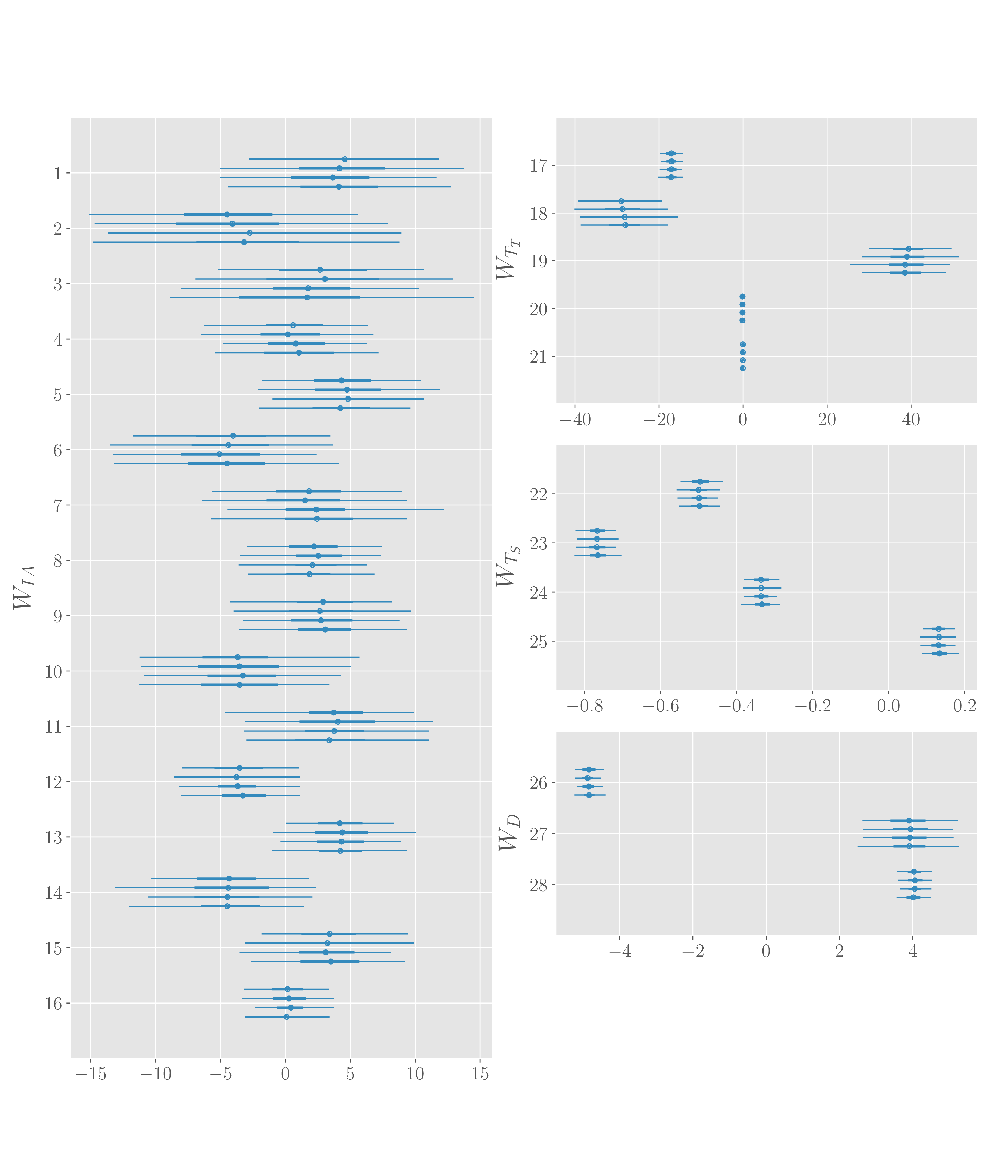

Supplement: S3 Fig — For each of four Markov chains, the mean (dot), the range from the 25% to 75% percentile (thick horizontal lines) as well as the 2.5% to 97.5% percentile (thin horizontal lines) are shown. For all parameters, these summary statistics of the marginal distribution are similar across all four chains, indicating convergence of the MCMC sampling scheme (see also S7 Fig). (TIFF) [file pone.0225838.s003.tiff]

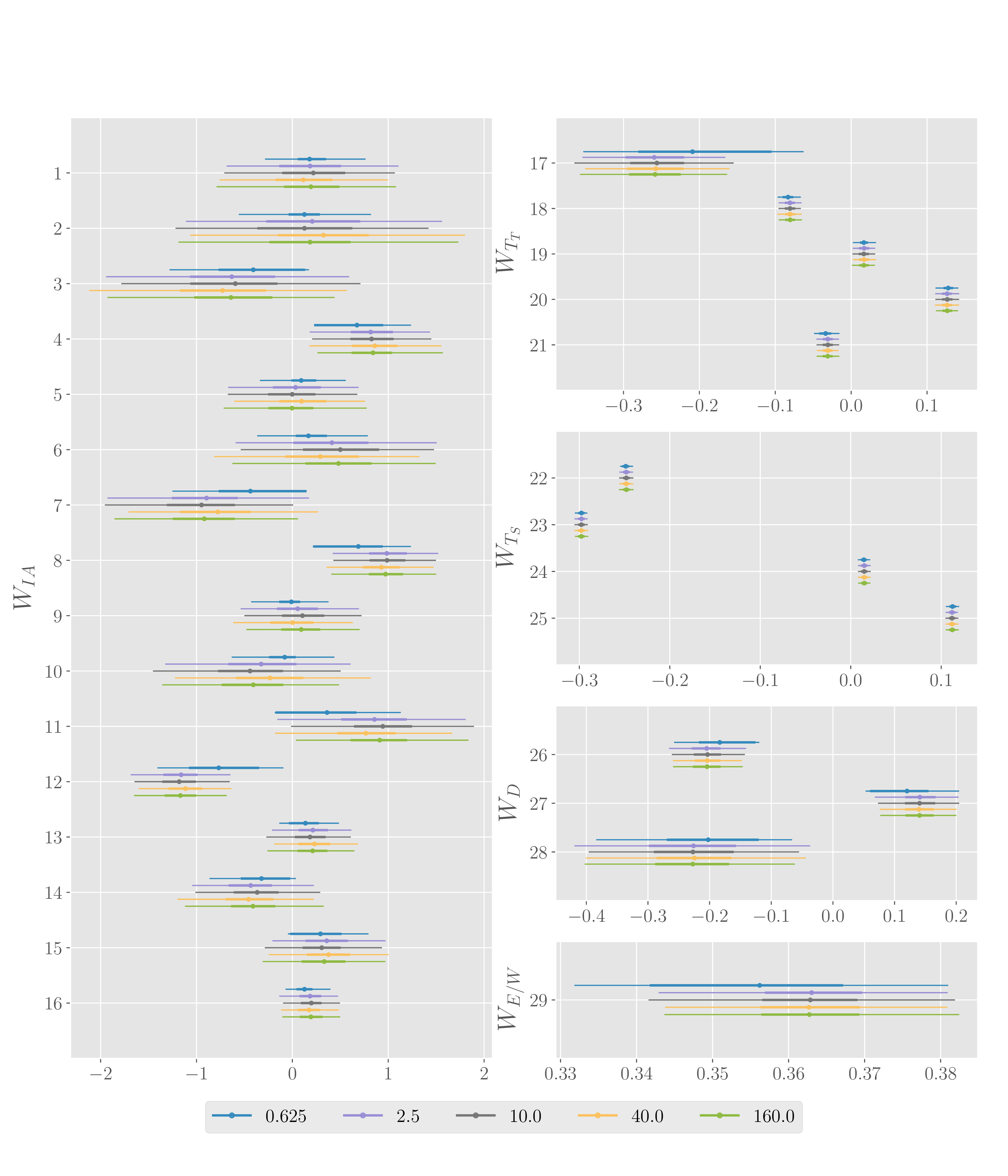

Supplement: S4 Fig — Marginal posterior distributions of all parameters are shown for five different scales σWIA={0.625,2.5,10.0,40.0,160.0} (color coded), which includes the special case σWIA=10 (see also S1 Fig) as used throughout this paper. For priors with standard deviation larger than 2.5, there is little qualitative change in the posterior distribution. (TIFF) [file pone.0225838.s004.tiff]

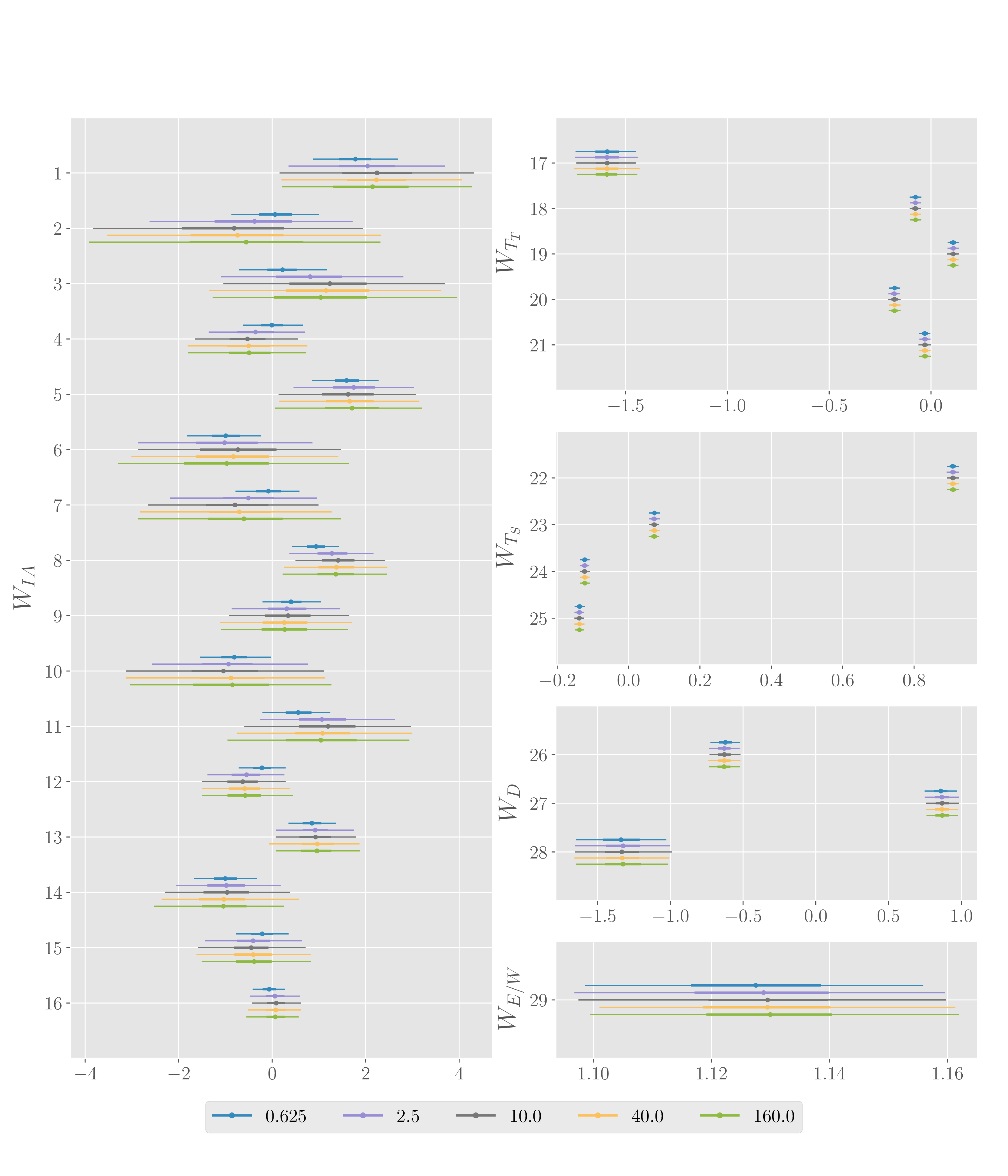

Supplement: S5 Fig — Marginal posterior distributions of all parameters are shown for five different scales σWIA={0.625,2.5,10.0,40.0,160.0} (color coded), which includes the special case σWIA=10 (see also S2 Fig) as used throughout this paper. For priors with standard deviation larger than 2.5, there is little qualitative change in the posterior distribution. (TIFF) [file pone.0225838.s005.tiff]

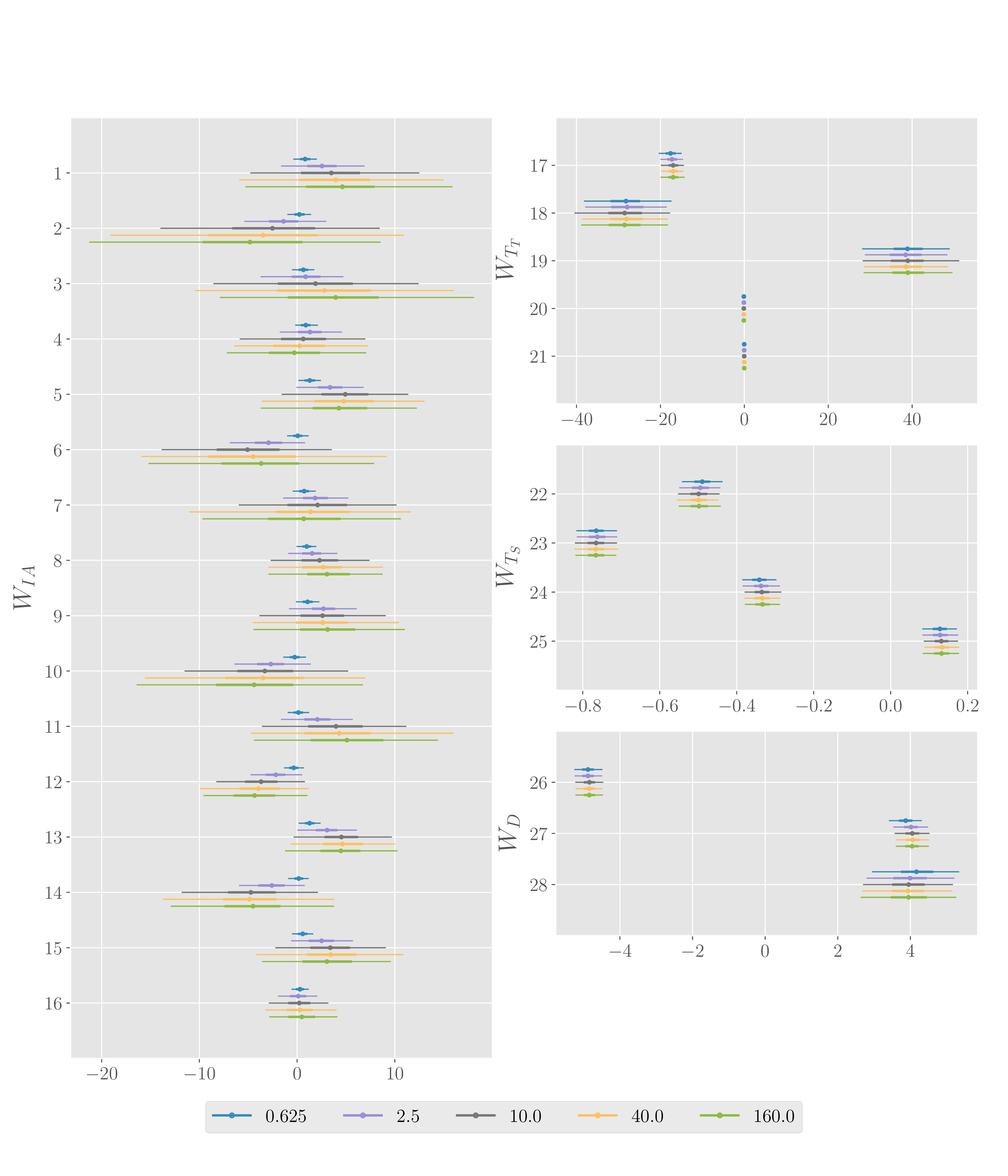

Supplement: S6 Fig — Marginal posterior distributions of all parameters are shown for five different scales σWIA={0.625,2.5,10.0,40.0,160.0} (color coded), which includes the special case σWIA=10 (see also S3 Fig) as used throughout this paper. Here, the choice of prior has considerably more impact on the posterior distribution than for campylobacteriosis (see S4 Fig) or rotavirus (see S5 Fig), for both of which more training data is available. For a narrow prior with standard deviation 0.625, the interaction effect coefficients appear to be strongly regularized towards zero. (TIFF) [file pone.0225838.s006.tiff]

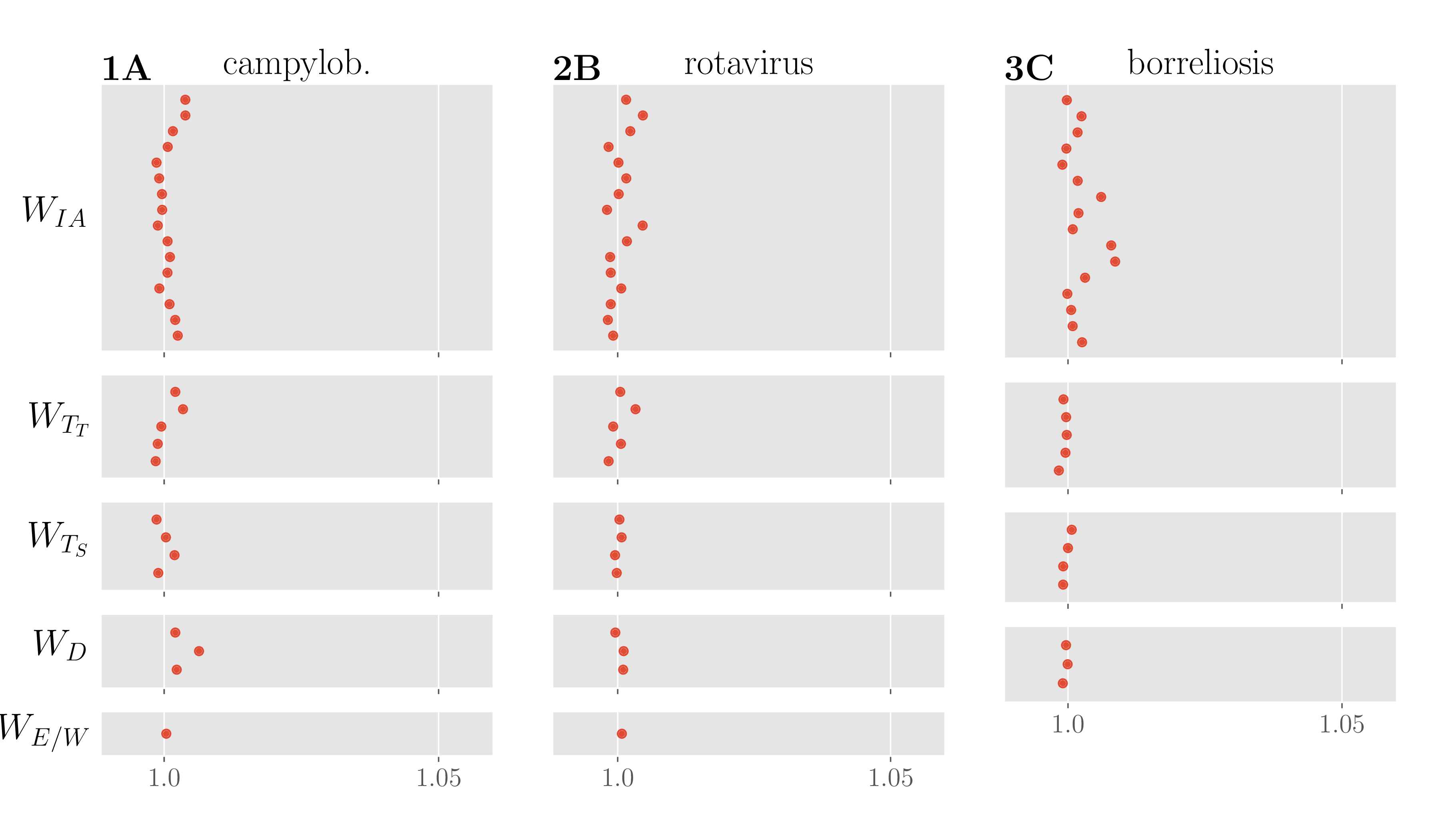

Supplement: S7 Fig — Gelman-Rubin diagnostics (red dots) for all parameters for campylobacteriosis (1A), rotavirus (2B) and borreliosis (3C). The values all lie close to 1.0 for all parameters, indicating convergence of the sampling procedure. (TIFF) [file pone.0225838.s007.tiff]

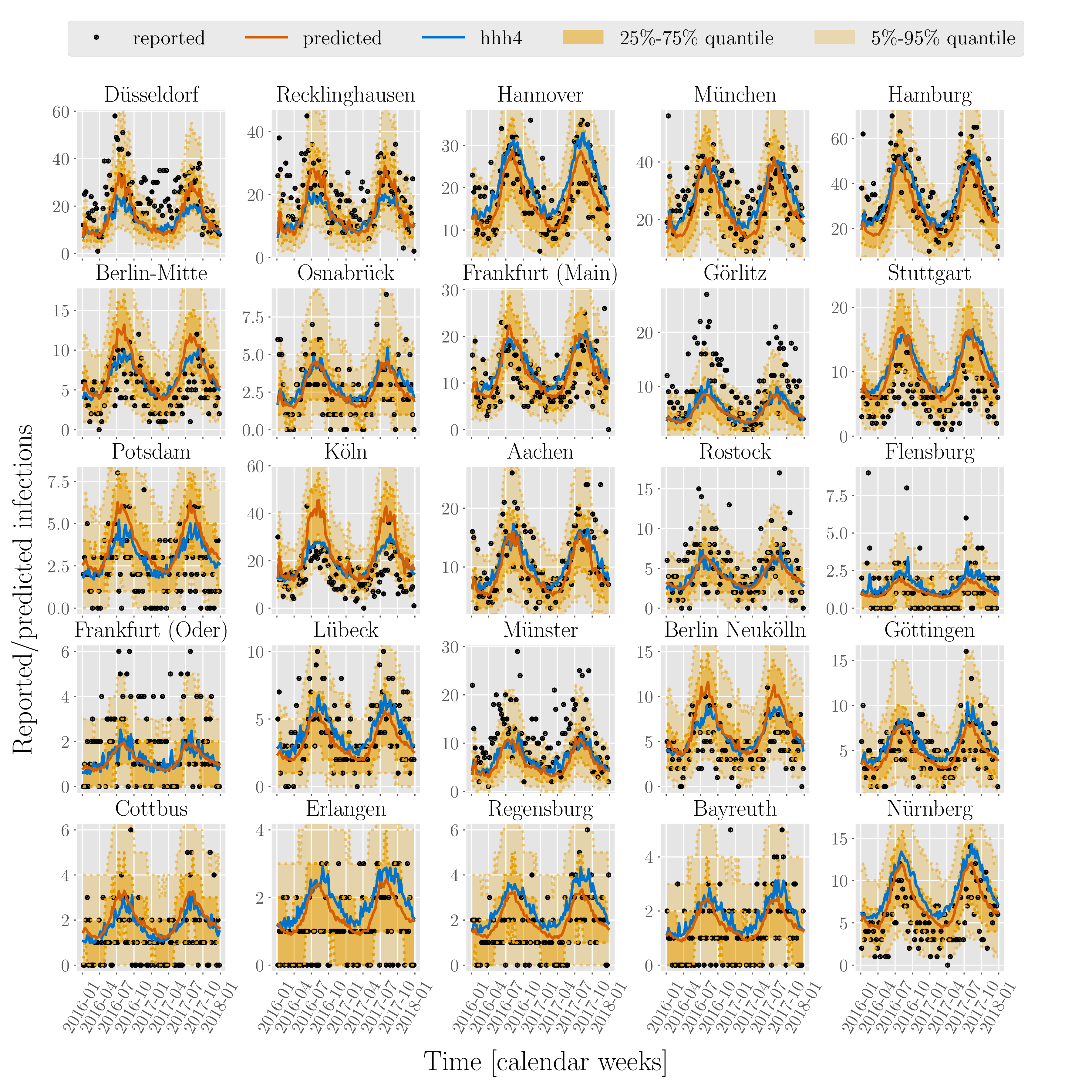

Supplement: S8 Fig — Reported infections (black dots), predictions of case counts by BSTIM (orange line) and the hhh4 reference model (blue line) for campylobacteriosis for 25 counties in Germany. The shaded areas show the inner 25%-75% and 5%-95% percentile. (TIFF) [file pone.0225838.s008.tiff]

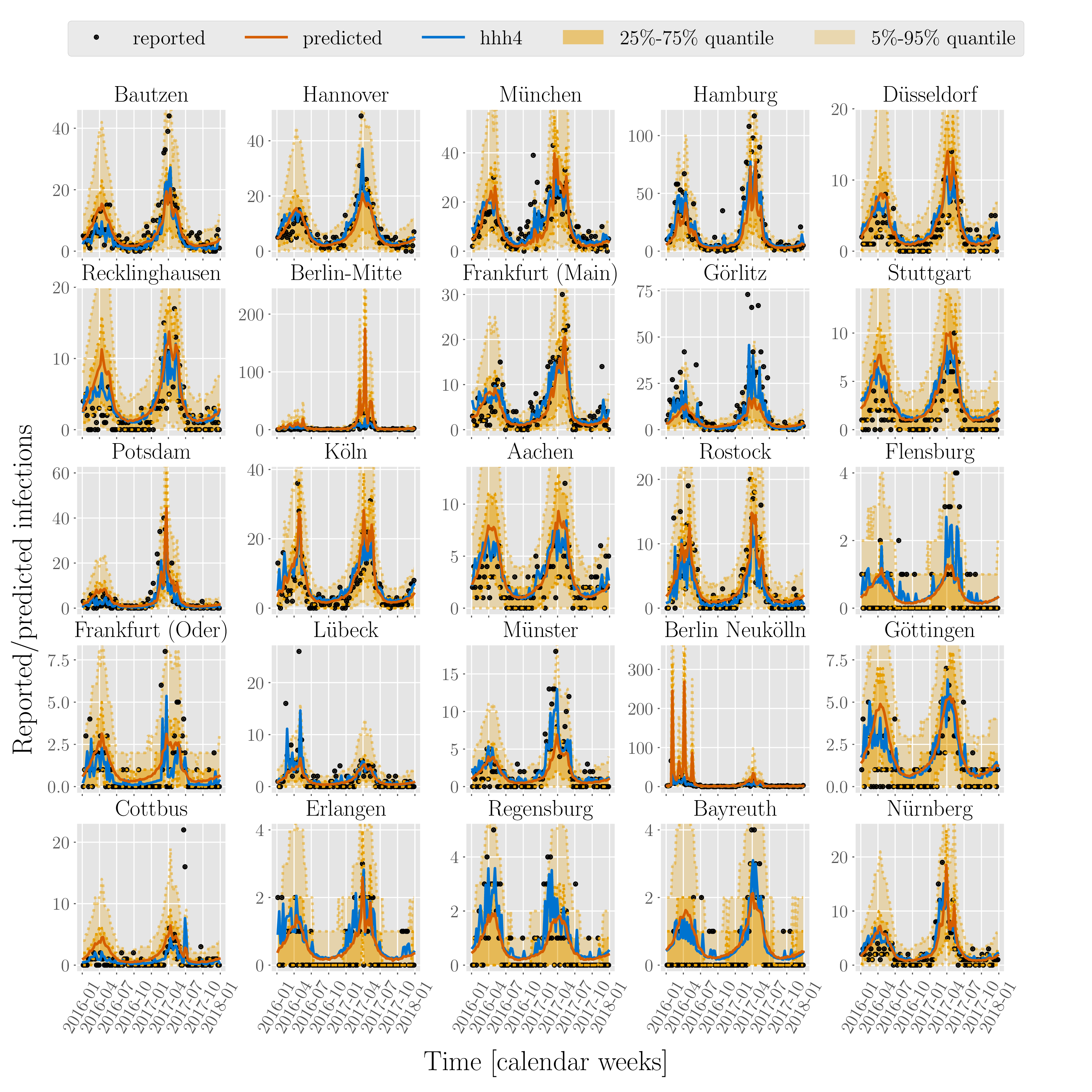

Supplement: S9 Fig — Reported infections (black dots), predictions of case counts by BSTIM (orange line) and the hhh4 reference model (blue line) for rotavirus for 25 counties in Germany. The shaded areas show the inner 25%-75% and 5%-95% percentile. (TIFF) [file pone.0225838.s009.tiff]

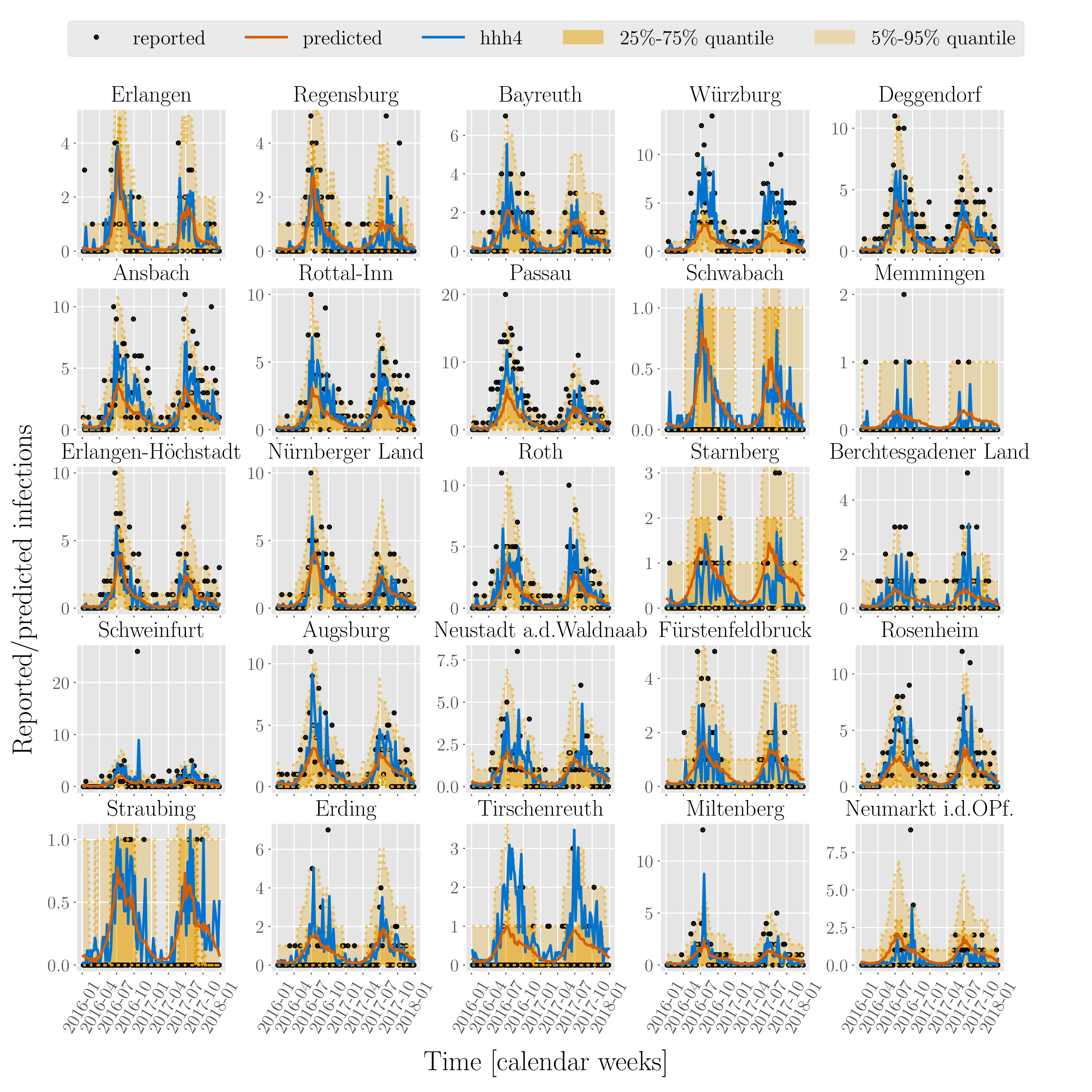

Supplement: S10 Fig — Reported infections (black dots), predictions of case counts by BSTIM (orange line) and the hhh4 reference model (blue line) for borreliosis for 25 counties in Bavaria. The shaded areas show the inner 25%-75% and 5%-95% percentile. (TIFF) [file pone.0225838.s010.tiff]
